# Genetic Map of Triticale Integrating Microsatellite, DArT and SNP Markers

**DOI:** 10.1371/journal.pone.0145714

**Published:** 2015-12-30

**Authors:** Mirosław Tyrka, Dorota Tyrka, Maria Wędzony

**Affiliations:** 1 Department of Biochemistry and Biotechnology, Faculty of Chemistry, Rzeszow University of Technology, Rzeszow, Poland; 2 Institute of Biology, Faculty of Geography and Biology, Pedagogical University of Krakow, Krakow, Poland; 3 Institute of Plant Physiology Polish Academy of Sciences, Krakow, Poland; New Mexico State University, UNITED STATES

## Abstract

Triticale (×Triticosecale Wittm) is an economically important crop for fodder and biomass production. To facilitate the identification of markers for agronomically important traits and for genetic and genomic characteristics of this species, a new high-density genetic linkage map of triticale was constructed using doubled haploid (DH) population derived from a cross between cultivars ‘Hewo’ and ‘Magnat’. The map consists of 1615 bin markers, that represent 50 simple sequence repeat (SSR), 842 diversity array technology (DArT), and 16888 DArTseq markers mapped onto 20 linkage groups assigned to the A, B, and R genomes of triticale. No markers specific to chromosome 7R were found, instead mosaic linkage group composed of 1880 highly distorted markers (116 bins) from 10 wheat chromosomes was identified. The genetic map covers 4907 cM with a mean distance between two bins of 3.0 cM. Comparative analysis in respect to published maps of wheat, rye and triticale revealed possible deletions in chromosomes 4B, 5A, and 6A, as well as inversion in chromosome 7B. The number of bin markers in each chromosome varied from 24 in chromosome 3R to 147 in chromosome 6R. The length of individual chromosomes ranged between 50.7 cM for chromosome 2R and 386.2 cM for chromosome 7B. A total of 512 (31.7%) bin markers showed significant (P < 0.05) segregation distortion across all chromosomes. The number of 8 the segregation distorted regions (SDRs) were identified on 1A, 7A, 1B, 2B, 7B (2 SDRs), 5R and 6R chromosomes. The high-density genetic map of triticale will facilitate fine mapping of quantitative trait loci, the identification of candidate genes and map-based cloning.

## Introduction

Hexaploid triticale (×Triticosecale Wittm.) with a genomic constitution of 2n = 6x = 42 (AABBRR) is an important cereal cultivated worldwide on about 4 million ha and over 70% of production is concentrated in European Union [1]. Commercial importance of this crop is justified as triticale combines favourable agronomic characteristics of wheat (i.e., high yield potential and good grain quality) and rye (i.e., abiotic stress tolerance). Breeding programs of triticale target improvement of the bread-making properties [2], adaptation towards biotic and abiotic stresses [3–4], and development of hybrid cultivars [5–9]. Recently, triticale has also been considered as bioenergy crop [7,10].

Application of genetic DNA markers is indispensable part of modern plant breeding. Identification of markers linked or located within target genes determining valuable traits influence selection efficiency. There are two main, complementary approaches for identification of target genes i.e. functional and positional cloning. Genetic maps obtained with the use of high throughput methods employing next generation sequencing are prerequisite for the positional cloning approach and identification of markers closely related to effects in genome wide association (GWAS) or precise localization of quantitative trait loci (QTLs). The high-density reference maps provide also a useful resource for gene mapping and linking physical and genetic maps, comparative genomics, as well as for predicting total breeding and genetic values for traits of agricultural significance [11–13].

One of the first high throughput marker system adopted to study numerous species was hybridization based DArT system [14]. DArT markers have already been developed for wheat [15], rye [16] and triticale [17]. Several genetic maps integrating DArTs with various PCR markers have been created for wheat [11,18–21], rye [9,16], and triticale [22].

New sequencing technologies emerged to meet demands of modern human diagnostic and were adapted for fast and cost-efficient genotyping and sequencing of plant genomes. At present, next-generation sequencing (NGS) is frequently used for the discovery of single-nucleotide polymorphisms (SNPs). However, presence of repetitive sequences accounting respectively for >80% and 92% of wheat and rye genomes [23,24] reduces number of unique SNP scoring. Two main approaches are used to reduce complexity of genomic sequences. First uses methylation sensitive restriction enzymes additionally selected to avoid species specific repetitive sequences [15,25,26]. A set of methods using restriction enzyme for complexity reduction called restriction site-associated DNA sequencing (RAD-seq), complexity reduction of polymorphic sequences (CRoPS), genotyping-by-sequencing (GBS), sequence-based genotyping (SBG), and DArTseq was developed [19,27–29] and applied for constructing genetic maps of crops with large genomes [19,26,30]. The second method of complexity reduction uses preselection of predefined fraction of complex genome most interesting for custom application [31–33].

The objectives of this study were (i) to develop a high density genetic map for hexaploid winter triticale compiling SSR (simple sequence repeat), and DArT (Diversity Arrays Technology) markers, and SNP markers revealed with DArTseq technology using a doubled haploid (DH) population, (ii) to analyze the extent of segregation distortion, and (iii) evaluate the quality of linkage map by comparing with previously reported maps that can lead to identification of structural rearrangements in triticale versus wheat and rye genomes.

## Materials and Methods

### Plant material

A population of 92 DH lines was developed from an intervarietal cross between two hexaploid winter triticale cultivars. ‘Hewo’ registered since 2001 by Strzelce Plant Breeders Ltd (Plant Breeding and Acclimatization Institute Group, Poland) was used as the female parent and the cultivar ‘Magnat’ registered since 2000 by Danko Plant Breeders Ltd, Poland was the pollen donor. These genotypes were chosen as parents for map construction because of their different frost tolerance, lodging resistance, preharvest sprouting tolerance, and reaction to *Microdochium nivale* [34]. The hybrid plants were kept under greenhouse conditions until flowering. Several F_1_ plants were used to develop the DH lines by using the maize method [35] with modifications [22].

### DNA isolation/extraction

Total genomic DNA was isolated from bulk of 5–15 young leaves per genotype as previously described [36]. DNA integrity was tested on agarose gels, while its quantity was measured spectrophotometrically.

### Simple Sequence Repeats

Simple Sequence Repeats (SSR) analyses comprised 45 markers that were polymorphic between the two parental lines. The selected assays included 25 wms [37], 7 scm [38,39], 3 gdm [40] 2 barc, and 8 wmc primers [41]. Amplification by PCR was performed following the protocol described previously [37]. The amplified fragments were separated by electrophoresis on 4% polyacrylamide denaturing gels and visualized using the silver staining method [42].

Genomic DNA from doubled haploid lines and their parents was sent to Diversity Array Technology (Yarralumla, Australia) for profiling using triticale high resolution array (DArT) with 7296 probes representing markers from wheat, rye, and triticale (wPt, rPt, and tPt, respectively). The samples were also used for genotyping by sequencing (Triticale GBS 1.0) service. GBS analysis results are split into biallelic dominant silicoDArTs markers and codominant DArTseq markers. Each marker scores for each sample were converted into “A” (Hewo), “B” (Magnat), and “-” (missing data) by comparison against parental scores.

### Construction of the genetic map

Markers of unknown parental origin and present at frequency 95%<F<5% were removed from the dataset. To simplify calculations, DArTs, DArTseq and silicoDArT markers were binned with QTL IciMapping [43] and representative markers with the lowest number of missing data were left to represent bins. Segregation data were analysed with JoinMap 4 [44] to group markers using a logarithm of odds (LOD) > 4. Then, markers within these groups were recurrently ordered using JoinMap maximum likelihood option and the RECORD program [45]. Group length and maximum expected number of recombinations per individual were criteria for selecting marker order for next ordering cycle. The better marker order was used to sort markers within linkage groups, and graphical genotypes were examined in Excel 2013. At this step, singletons (a single locus in one progeny line that appears to have recombined with both its directly neighbouring loci) were replaced by missing values in the dataset and calculations were repeated until no singletons were found (no more than three rounds).

### Comparative analysis of genetic map

*De novo* mapping approach was used to construct genetic map of ‘Hewo’ × ‘Magnat’ DH population. Resulting marker order corresponding to chromosomes representing A and B genomes of wheat was compared with respective reference marker according to positions on wheat consensus map provided by Diversity Arrays Technology (unpublished). DArT and SSR markers were used to assign linkage groups to chromosomes from rye genome. Order of markers in groups corresponding to rye was compared to map of rye [16] and consensus triticale map [46].

Details of where experiment occurred: Mapping population was developed at Institute of Plant Physiology Polish Academy of Sciences in Krakow. SSR and DArT analyses were completed at Centre of Applied Biotechnology, Polonia University in Czestochowa (this laboratory is closed now). The first calculations of the genetic map (for DArT and SSR markers) were started at Rzeszow University of Technology (RUT), and finished at 2012. Next, DArTseq markers were developed at RUT and whole map was recalculated at the beginning of 2015.

## Results

### Markers distribution

Segregations of markers were recorded for 89 double haploid lines of ‘Hewo’ × ‘Magnat’ population (DH-HM). Initially, 50 simple sequence repeat (SSR), 963 Diversity Arrays Technology (DArT) and 16888 DArTseq markers were used to assemble the genetic map. About 87% of DArT and 80% of DArTseq markers (842 and 13452, respectively) were assigned to the 20 chromosomes, covering 4907.4 cM, with a mean distance of 3.0 cM between adjacent markers. Additionally, 1880 highly distorted markers were assembled into structurally mosaic linkage group that not corresponded to missing chromosome 7R (Table 1).

**Table 1:** Distribution of markers on linkage groups of ‘Hewo’ × ‘Magnat’ population.

Chromo some	Length (cM)	Number of markers									Mean density*
		SSR	DArT			SNP-DArT	silicoDArT	Bin markers	Total	Distorted	
			wPt	tPt	rPt						
1A	301.6	2	23	2	0	210	418	122	655	225	2.5
2A	271.8	3	15	0	0	153	464	69	635	8	4
3A	203.4	1	17	3	0	57	307	51	385	22	4.1
4A	248	3	27	1	3	81	285	71	400	27	3.5
5A	344.3	4	9	4	0	163	282	109	462	109	3.2
6A	76.5	1	4	1	0	46	130	25	182	27	3.2
7A	382.2	2	31	6	0	248	728	115	1015	207	3.4
1B	237.3	0	57	7	0	178	603	92	845	796	2.6
2B	366	2	21	5	4	100	495	122	627	178	3
3B	353.4	3	42	6	0	119	467	96	637	7	3.7
4B	122.8	4	10	3	0	85	156	48	258	49	2.6
5B	248.5	4	15	2	0	79	293	61	393	99	4.1
6B	278.5	2	43	11	1	104	561	101	722	263	2.8
7B	386.2	3	42	5	0	237	751	141	1038	613	2.8
1R	151.4	0	0	3	44	20	245	41	312	95	3.8
2R	50.7	0	0	3	24	47	255	29	329	5	1.8
3R	60.9	0	0	1	30	45	380	24	456	1	2.6
4R	193.7	1	3	8	99	77	446	52	634	318	3.8
5R	247.1	8	1	5	76	257	1456	99	1803	1356	2.5
6R	383.1	7	0	12	113	341	2033	147	2506	755	2.6
A genome	1827.8	16	126	17	3	958	2614	562	3734	625	3.3
B genome	1992.7	18	230	39	5	902	3326	661	4520	2005	3.0
R genome	1086.9	16	4	32	386	787	4815	392	6040	2530	2.8
Total	4907.4	50	360	88	394	2647	10755	1615	14294	5160	3.0
Mosaic group	298.8	0	11	3	2	360	1504	116	1880	1880	2.6

*The mean density equal to L/(n-1) where n is the number of unique markers per chromosome length (L)

To simplify calculations the total number of markers was reduced by 89% from 16174 to 1731 bin markers representing up to 322 shortlisted loci. In total the highest saturation with markers was found for genome R, then B and A, however taking into account unique markers, six chromosomes of genome R showed the lowest number of bin markers followed by markers from genomes B and A. This is related with different binning ratio: for genomes A and B about 14.6–15% of markers were unique, while for R genome and mosaic linkage group only 6.2–6.5% of markers showed unique segregation pattern. The higher binning efficiency may be related to suppressed recombination in case of chromosomes from R genome in spite of the highest diversity available in this genome (Table 1).

Assuming equal chance for not specific hybridization, distribution of DArT markers derived from wheat (wPt), rye (rPt), and triticale (tPt) reflects level of genetic rearrangements. Genomes A and B contained 420 DArT markers of type wPt (84.8%), rPt (1.9%), and tPt (13.3%). Genome R consisted of 91.5% rPt, 0.9% wPt and 7.6% of tPt DArTs accounting for total number of 422 markers. Higher share of tPt markers in linkage groups of A and B genomes indicate that these genomes are modified in higher rate in triticale than chromosomes from genome R (Table 1).

### Genetic map

**Fig 1:**
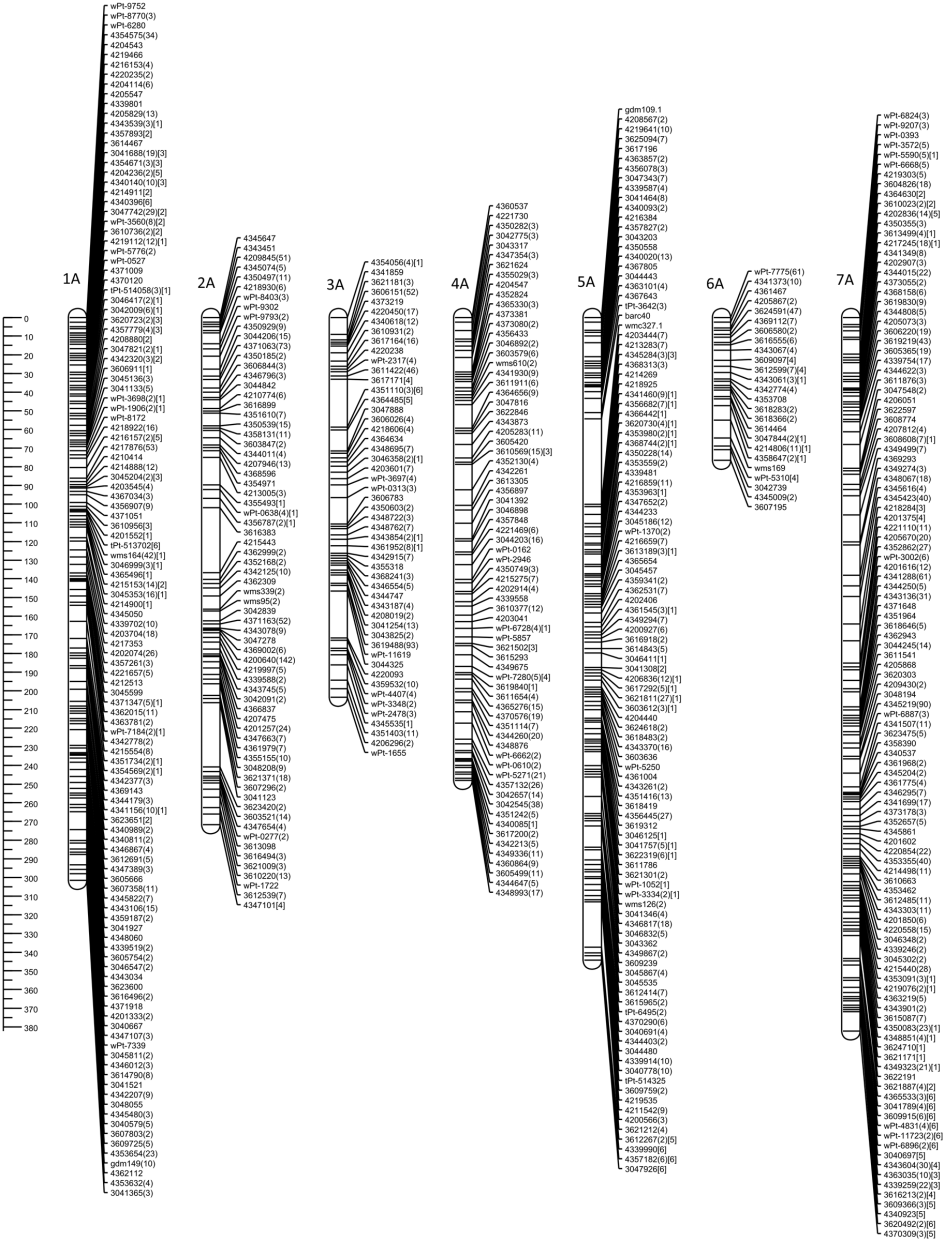
Genetic linkage map of the triticale A genome obtained with 89 doubled haploid lines of the ‘Hewo’ × ‘Magnat’ population comprising SSR, DArT, and DArTseq markers. Numbers in parentheses indicate the total number of markers represented by bin marker. Segregation distortions of markers are indicated by number in square parentheses at significance levels and corresponding respectively to p< 0.05 [1], p< 0.01 [2], p< 0.005 [3], p< 0.001 [4], p< 0.0005 [5], and 0.00001 [6]. The ruler on the left indicates distances in centiMorgans (Kosambi).

**Fig 2:**
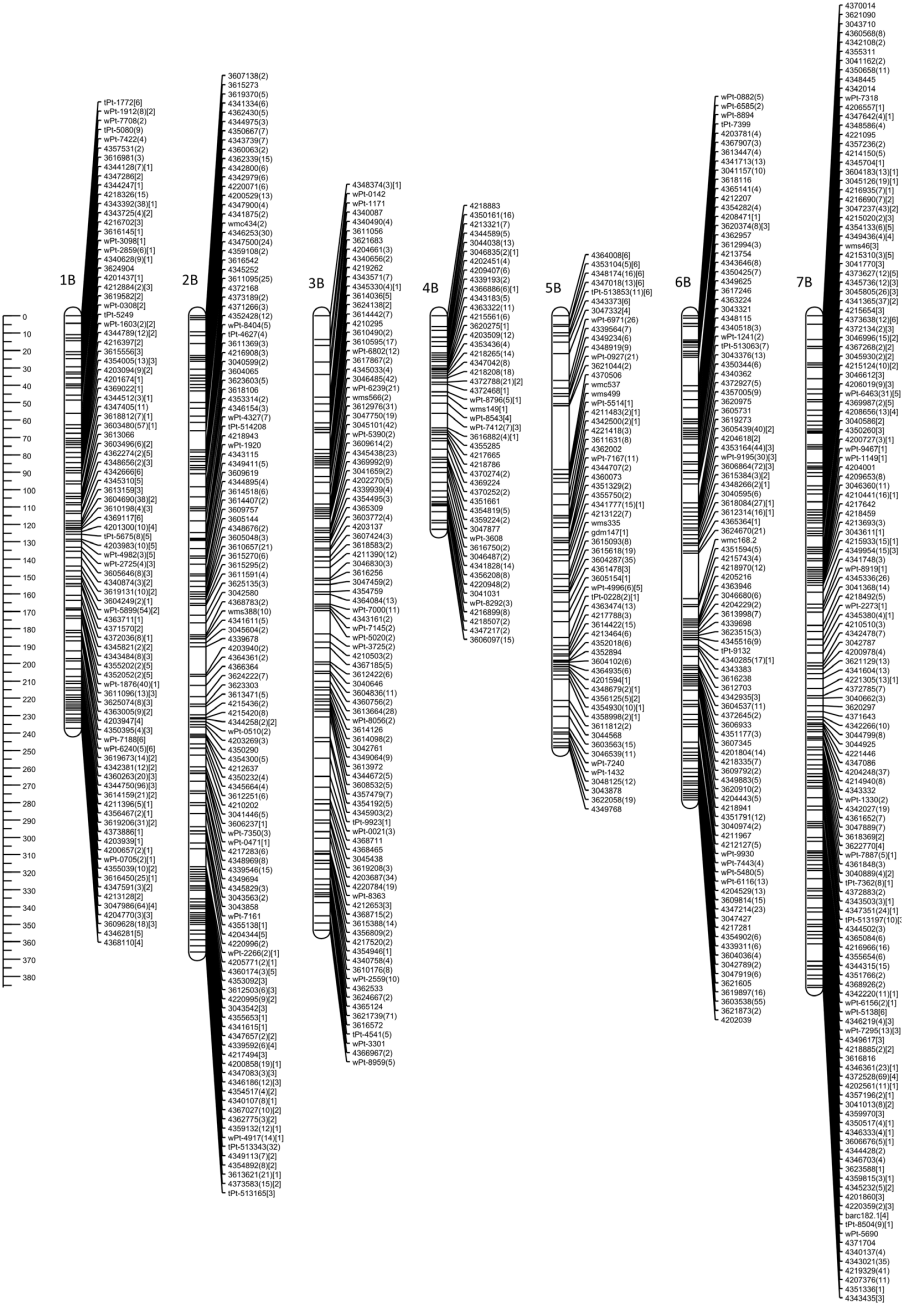
Genetic linkage map of the triticale B genome obtained with 89 doubled haploid lines of the ‘Hewo’ × ‘Magnat’ population comprising SSR, DArT, and DArTseq markers. The ruler on the left indicates distances in centiMorgans (Kosambi).

**Fig 3:**
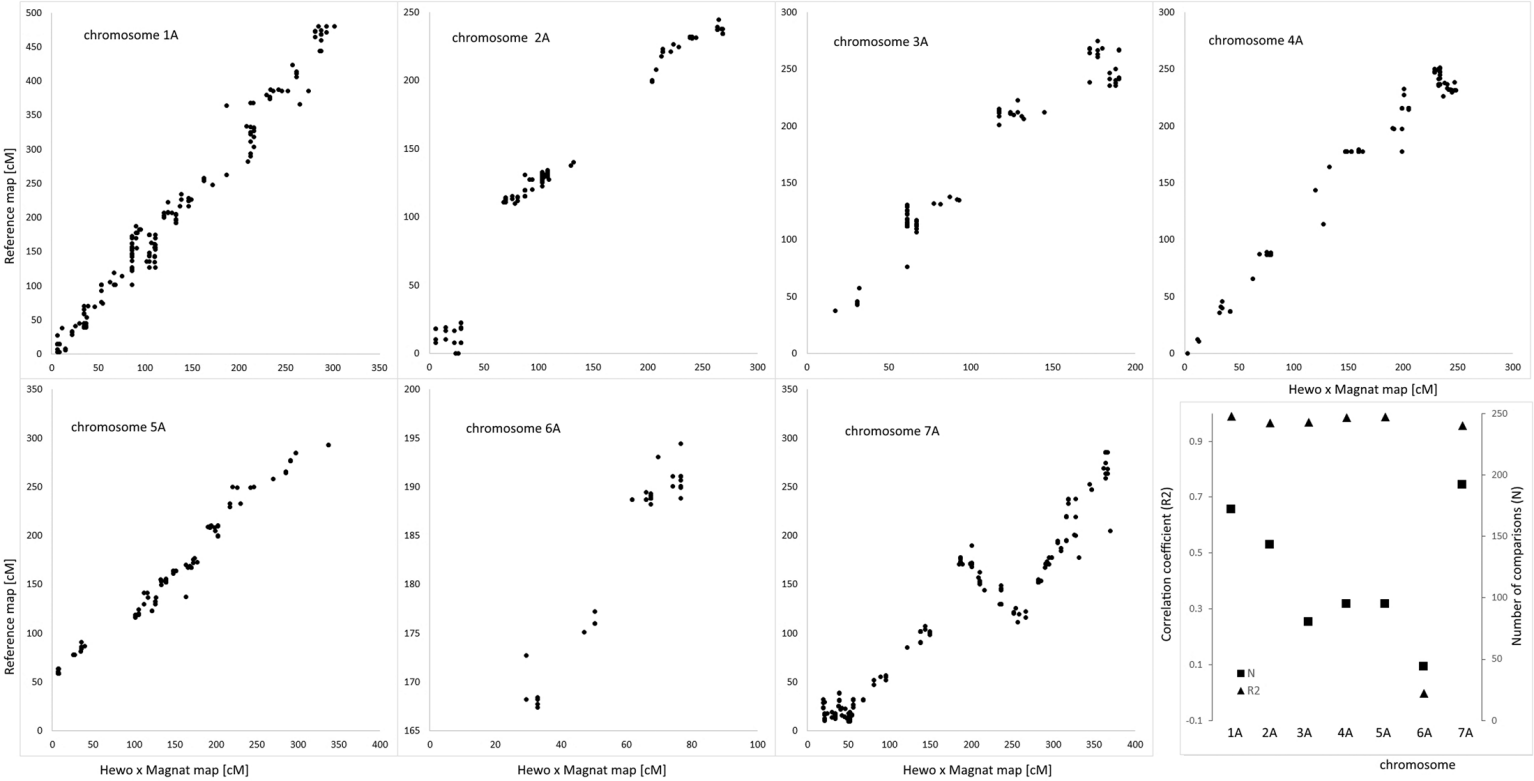
Comparative distribution of markers on consensus reference wheat map and linkage groups from A genome of HM-DH population.

**Fig 4:**
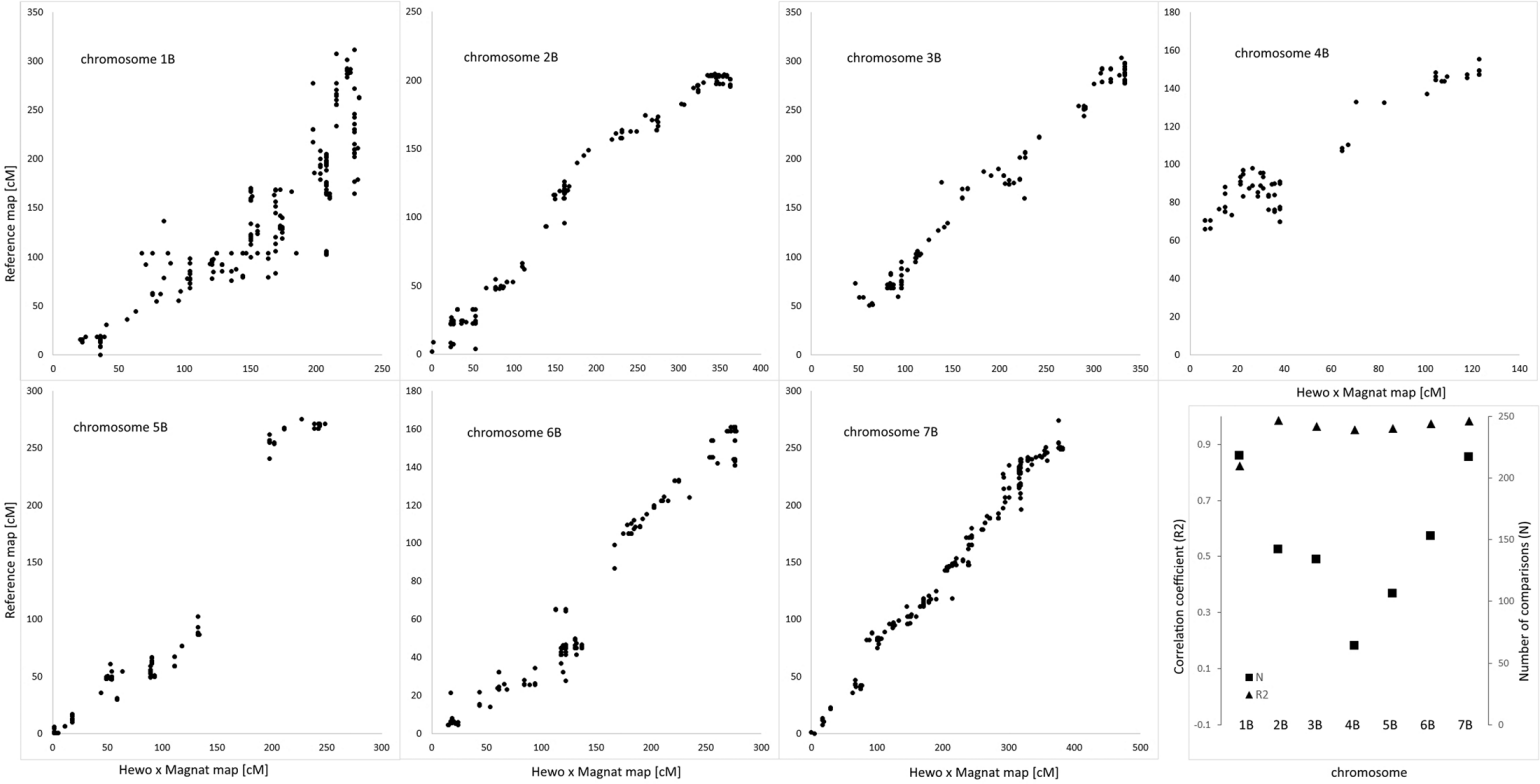
Comparative distribution of markers on consensus reference wheat map and linkage groups from B genome of HM-DH population.

The genetic map of DH-HM showed good coverage of the A and B genome chromosomes, apart from 6A. Compared with positions on consensus map of wheat, most shared markers showed highly consistent orders with correlation coefficient exceeding 0.900. The maps in Figs 1 and 2 include representations of all markers assigned to the identified linkage groups, grouped by genome. To simplify the map, redundant co-located markers were represented by a single, representative bin marker with the total number of markers represented (S1 Table) given in parentheses after the marker name. Genetic map was constructed for bin markers and redundant markers were added to saturate the map and to supplement sequence information (S2 and S3 Tables). Loci of DArTseq markers on chromosomes from the A and B genomes were compared with reference positions for wheat provided within service (Figs 3 and 4).

Map length of the A genome (1827.8 cM) was shorter than that of the B genome (1992.7 cM). All chromosomes in genomes A and B were represented by single groups. However, gaps exceeding 30 cM were present in chromosomes 2A (34.8, and 34.5 cM), 5A (45.7 cM) and 5B (36.6 cM) (Figs 1 and 2). The average length of linkage groups of A and B genomes was 272.9 cM, with a maximum of 386.2 cM, for 7B, and a minimum of 76.5 cM, for 6A. The average distance between markers on chromosomes of A genome ranged from 2.5 cM for 1A to 4.1 cM for 3A, and 5B (Table 1).

Generally, all triticale chromosomes from the A and B genomes remained collinear with reference wheat map for DArTseq markers. In genome A no structural modification was found in chromosomes 1A, 2A, 3A, and 4A. Chromosome 5A of MH-DH missed 50 cM of distal part of short arm. Fragment of chromosome 6A of HM-DH corresponds to 30 cM fragment starting from 165 cM of respective reference wheat chromosome. High density marker comparison allowed to reveal inversion in proximal fragment of linkage group 7A. In genome B no rearrangements were found, but 4B chromosome with missing region of distal fragment of short arm equivalent to 65 cM of reference wheat map. Colinearity of chromosomes from genome B measured as correlation was 0.82 for 1B chromosome, while for remaining exceeded 0.95 (Figs 3 and 4).

**Fig 5:**
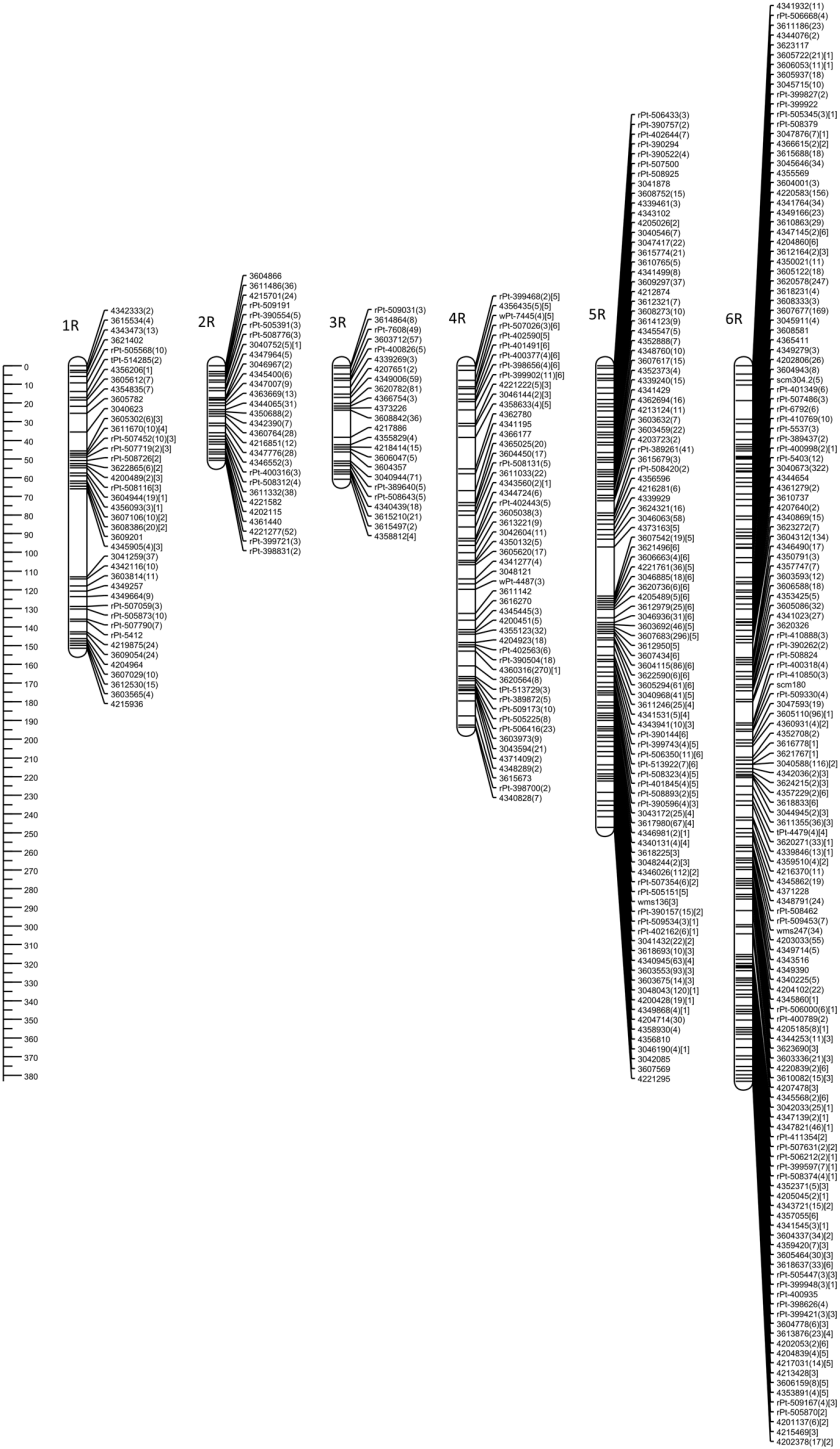
Genetic linkage map of the triticale R genome obtained with 89 doubled haploid lines of the ‘Hewo’ × ‘Magnat’ population comprising SSR, DArT, and DArTseq markers.

**Fig 6:**
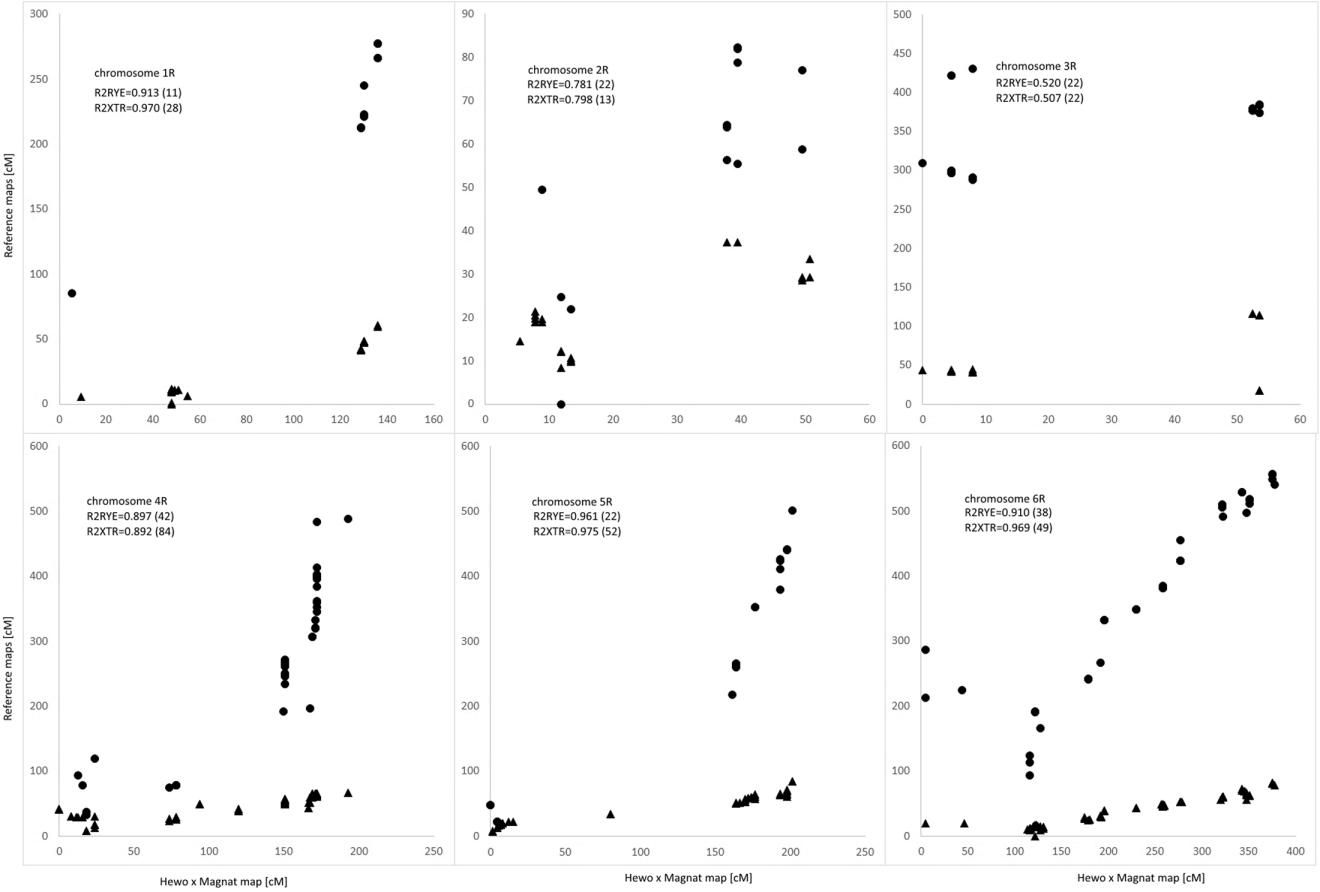
Comparative distribution of markers on consensus reference wheat map and linkage groups from A genome of HM-DH population.

R genome of HM-DH population consisted of 6 linkage groups with 7R chromosome missing (Fig 5). Chromosome 1R consisted of two clusters of markers corresponding to short and long arm put into single chromosome at distance of 47.1 cM. DArTseq markers for R genome are not annotated with chromosome position. Then, localization of DArT markers on consensus map of triticale [46] and rye [16] was used to identify linkage groups (Fig 6). Chromosomes 1R, 4R, 5R and 6R showed good agreement with rye and triticale maps expressed by high correlation coefficients (0.89–0.98). Fragment of chromosome 1R of HM-DH equal to 7cM corresponds to 53 cM region in the genetic map of rye (from 213 to 266 cM). Colinearity of markers from chromosomes 2R and 3R was low (0.78, and 0.52, respectively). These linkage groups were the shortest in R genome of HM-DH population (50.7 and 60.9, respectively) with markers of suppressed recombination in respect to rye genetic map. Suppressed recombination in triticale vs rye is also well visible in case of 4R and 5R where in rye markers spanning 300 cM are packed in 50 cM fragments of HM-DH population.

Positions of loci of HM-DH population vs. triticale [46] are marked with triangles and vs. rye map [16] with circles.

### Segregation distortion

A χ2 test was performed to verify the adjustment of every marker to the expected Mendelian segregation ratio in doubled haploids. Significant deviation (p < 0.05) from the expected 1:1 ratio was found for 5160 (36%) out of the 14294 markers. Distorted segregation was not taken as criterion to eliminate markers. Alleles of female parent were overrepresented in the genotypic data collected in this study, and 49.4% of the alleles in the HM-DH population were derived from ‘Hewo’, 44.3% were from ‘Magnat’, and the remaining 6.3% were missing data. Distorted markers were distributed in 30 regions mapped on 17 of the 20 triticale chromosomes with exception of 4A, 2R, and 3R (Table 2). Although, these regions were equally shared between the two parents, for multiple distortion regions present on chromosomes 6A, 5B, 7B, 6R single parent was preferred. In case of chromosomes 5A and 7A distortion regions present on distal fragments were biased to ‘Hewo’ while in remaining regions alleles coming from ‘Magnat’ were preferred. Main segregation distortion regions exceeding 1% of total number of bin markers equal to 17 markers were present at 1A, 7A, 1B, 2B, two regions on 7B, 5R and 6R (Table 2).

**Table 2:** Distribution of markers in 30 segregation distorted chromosomal regions (SDR). Numbers in parentheses indicate the total number of markers represented by bin marker.

SDR	Chrom.	Position	Flanking markers	Distorted markers		
				Bin	Total	Source
1	1A	16.7–112.3	4343539(3)—4214900	51	330	Magnat
2	2A	89.8–97.0	4355493–4356787(2)	3	7	Hewo
3	3A	33.0–60.3	3617171–4364485	3	5	Magnat
4	5A	120.8–132.4	4341460(9)—4368744(2)	6	25	Magnat
5	5A	179.8–194.3	3046411–3603612(3)	6	49	Magnat
6	5A	223.1–227.0	3046125–3622319(6)	3	12	Magnat
7	5A	337.3–344.3	3612267(2)—3047926	4	10	Hewo
8	6A	22.9–29.5	3609097–4343061(3)	3	11	Magnat
9	6A	42.3–47.1	3047844(2)—4358647(2)	3	15	Magnat
10	7A	22.8–34.3	4364630–4217245(18)	6	42	Magnat
11	7A	318.6–382.2	4350083(23)—4370309(3)	22	150	Hewo
12	1B	24.8–237.3	4344128(7)—4368110	85	816	Hewo
13	2B	297.9–366.0	4355138—tPt-513165	30	208	Hewo
14	3B	67.2–79.2	4345330(4)—3624138	3	6	Hewo
15	4B	38.1–64.6	4372788(21)—3616882(4)	7	40	Hewo
16	5B	0.0–21.8	4364008–3047332	7	48	Hewo
17	5B	95.8–108.7	wPt-5514–4342500(2)	3	5	Hewo
18	5B	164.6–185.1	4361478—tPt-0228(2)	4	10	Hewo
19	5B	201.8–209.1	4201594–4358998(2)	5	20	Hewo
20	6B	112.9–135.4	3605439(40)—4365364	11	242	Hewo
21	7B	65.3–141.0	4345704—wPt-1149	33	301	Magnat
22	7B	153.6–159.0	3043611–4349954(15)	3	31	Magnat
23	7B	252.5–271.8	3618369—tPt-513197(10)	10	61	Magnat
24	7B	300.0–371.2	4342220(11)- tPt-8504(9)	26	190	Magnat
25	1R	45.6–66.0	3605302(6)—4345905(4)	14	95	Hewo
26	4R	0.00–38.4	4358812–4358633(4)	13	47	Hewo
27	5R	97.1–221.1	4373163–4349868(4)	50	1351	Hewo
28	6R	68.8–74.9	4347145(2)—3612164(2)	3	5	Magnat
29	6R	202.1–241.8	3605110(96)—4359510(4)	16	319	Magnat
30	6R	275.9–383.1	4345860–4202378(17)	46	394	Magnat

## Discussion

### Saturation of triticale genome

The genetic map saturation depends on variation between two parents that can be detected in DNA fragments divided by recombination events (bins) in combinatorial sets limited by number of individuals. In HM-DH population, the overall distribution of markers was not homogeneous between the triticale genomes with R and A genomes being the most and least represented. Similar unequal distribution was observed previously for the AFLP, RAMP, RAPD, and SSR markers, with 50.7% of those detected from R genome [47]. In current studies, the observation that, the R genome showed the highest coverage with DArT markers, followed by the B genome and the A genome [22,46] was confirmed also using DArTseq markers. Thus, this phenomena is rather not dependent on platform or marker system but reflects general diversity deposited in these genomes.

DArTseq system was applied for sequence-based SNP detection and genotyping of a biparental triticale population. We identified 18775 SNPs between the two parents and finally mapped 13402 (71.4%) DArTseq markers pooled into 1459 bins based on common segregation in the set of 89 double haploid lines. Next generation sequencing approach based on DArT-Seq was used in mapping of common wheat, where 3.6 million marker were anchored including 24408 DArTseq markers [21]. DArTseq markers has much better genome coverage when compared to DArT markers that have been often reported to be clustered in gene-rich regions [20]. Simultaneous application of hybridization based DArT analysis and SSRs markers were required for proper assignment of the rye part of the genome. For integration of DArTseq markers we used DArT based framework map composed of 842 markers in 272 bins.

The obtained genetic map of triticale covering 4907.4 cM with 14294 markers consists of the 20 chromosomes and is the most saturated genetic map of triticale when compared to the previously published DArT-based maps composed of up to 2555 markers [22,46]. Integration of various markers into genetic maps resulted in variable length of maps of the hexaploid wheat genome, that ranged from 2260 cM to 5332 cM [20]. The increase of total length of the *de novo* map can be associated with genotyping errors in the GBS dataset [11]. Taking into account 1615 unique bin markers mapped in HM-DH population, map resolution/density increased only about three times when compared to 692 markers in ‘Saka’ × ‘Modus’ [22], or 6 mapping populations with mean 613 unique markers (range from 435–755) per cross [46]. Mean map density measured in HM-DH map for bin markers was limited to 3.0, and this is higher when compared to biparental maps reported for triticale 4.0–5.2 [22,46]. However integration of GBS markers increased potential maximum resolution counted for all markers to 0.34. Redundant markers may be rich source of sequences for identification of target contigs and candidate genes in QTL mapping, and are important for construction of consensus maps.

Map resolution measured by mean marker density was restricted mainly by the size of mapping population. Therefore, with available highly efficient methods of genotyping and number of up to 5 crossing-over per individual [48] the resolution of individual genetic map can be increased by the use of mapping population with accumulated recombination events (i.e. application of recombinant inbreed lines) or/and by increase of number of segregating individuals (F_2_ or DH mapping populations).

### Distorted segregation

Triticale gametogenesis may face difficulties owing to the coexistence of genomes from species with different structural and molecular genomic characteristics and reproductive systems—autogamous wheat and allogamous rye—each of which carry different genes [47]. Autoalloploidy, presence of chromosomal rearrangements, and environmental factors may also result in selection of favorable allelic combinations at gametic or zygotic level [20]. In case of genetic map consisting of 380 markers, segregation distorted regions (SDR) were defined as a clusters of at least three closely adjacent loci with deviated segregation [49]. We suggest that number of clustered loci for selection of SRDs should be more relative and limited to 1% of nonredundant markers. Taking into account these criteria for SDR identification, in HM-DH population 17 adjacent markers would be a threshold, and the number of SDR may be reduced from 30 (Table 2) to 8 most important regions on chromosomes 1A, 7A, 1B, 2B, 7B (2 regions), 5R, and 6R.

Segregation distortion (SD) is a commonly observed phenomenon, especially in mapping populations of DH lines [50–52]. In triticale DH lines SD may be observed for 20%-29.1% markers [22,47]. Distorted markers favoring the maternal line ‘Saka3006’ allele were preferentially mapped on 3B, 6B, 7B, 1R, 6R, and 7R. Distorted alleles of pollinating parent ‘Modus’ were found predominantly on the remaining chromosomes of the A and B genomes (except on 7A, 1B, and 4B) and on 4R [22]. Segregation distortion in the microspore derived populations resulted in clusters of distorted markers on chromosomes 2B, 3B, 1R, 4R, and 7R [46]. Many doubled-haploid populations show some SD that is attributed to survival in anther or microspore culture [46,47,53,54]. However, SDRs may be also randomly distributed throughout a genome [13]. In populations derived from the crossing of rye inbred lines, molecular markers with distorted segregation were located on chromosomes 1R, 4R, 5R, 6R, and 7R [38,49,55–57], and SD in these region in triticale may be not related with the process of DH lines production.

### Structural comparison of triticale genetic map

The accuracy of the de novo DH-HM genetic map was assessed by comparing it with the high-density wheat reference map and for R genome with rye DArT and triticale consensus maps [16,46]. Comparative analysis revealed putative deletions in chromosomes 5A, 6A, and 4B, and inversion in 7A. Presence of duplicated fragments of chromosomes corresponding to the same localization on reference maps provide indirect evidence of structural rearrangement, that need validation by use of fluorescent *in situ* hybridization with site specific probes. Cytogenetic analyses revealed the presence of substitutions and translocations within a homoeologous and nonhomoeologous wheat chromosomes in triticale [58–60]. Discrepant marker orders caused by chromosome inversions were observed in some regions of *Triticum turgidum* genetic map, mostly on chromosomes 1A and 2A [18]. In triticale structural mutations such as deletions of distal chromosomal fragments (1A, 7A) and translocations involving 2B and 6A chromosomes were suggested on the base of comparisons with reference maps of wheat [22]. Presence of rearrangements may influence effectiveness of GWAS in triticale.

Segregation distortion affects genetic map distances and ordering of loci. Excluding from further analyses markers which significantly (P ≤ 0.001) deviated from the expected ratio in a chi-square test can even result in complete chromosomes being absent from genetic maps [46]. In previous efforts on triticale mapping, missing chromosomes 1R, 2A, 2R and 7R were reported [22,46]. In spite of high saturation of DH-HM genetic map with genetic markers we did not found linkage group corresponding to chromosome 7R. Chromosome 7R showed highly distorted segregation in triticale [22] and rye [61]. However, in ‘Hewo’ × ‘Magnat’ population all distorted markers were retained, and we found group of 1880 linked markers sorted into 116 bins that formed ‘spurious group’ composed of markers representing mainly ‘Hewo’ alleles.

Spurious group was formed from markers detecting real chromosome segments. While silico-DArTs are extracted *in silico* from sequence data and the presence-absence of this type of markers is analogous to DArT markers from microarray platforms [12], SNP markers are biallelic and situation, that 0 means no signal (deletion) should be excluded. On the other hand 1880 markers from spurious group account for 11.6% of total markers that exceed the range of 0.2–1.4% of markers [15,17,62] prescribed to intravarietal heterogeneity. Obtained ‘spurious group’ have mosaic construction (S1 Fig) because in 78% of population solely ‘Hewo’ alleles were present, and segregations in 20 individuals are not sufficient for proper ordering of loci.

We may hypothesize that the ‘spurious group’ is B chromosome. In rye, B chromosomes are autonomously inherited independent of the host genome, they use their own mechanisms of mitotic or meiotic drive, are generated in response to interspecific hybridization, are derived from fragments of A chromosomes, are rich in gene-derived sequences with the largest parts corresponding to rye chromosomes 3R and 7R, accumulate large amounts of specific repeats and insertions of organellar DNA, and possess genes controlling their own transmission [63]. Additionally, B chromosomes do not pair with any of the standard A chromosomes at meiosis, and have irregular modes of inheritance [64,65]. B chromosomes mainly occur in outcrossing species and are very frequent in Gramineae family [66]. B chromosomes derived from rye may be present in triticale [67]. It is then possible that we first report statistical evidence of descent of B chromosomes in triticale mapping population.

## Conclusions

We constructed the first ultrahigh-density linkage map for the triticale genome. At present this is the most saturated map of triticale first involving GBS markers, that may serve for identification of qualitative trait loci for important agronomic and biochemical traits. Short sequences of GBS markers may be used directly for landing to wheat A and B genome contig sequences.

## Supporting Information

S1 Fig Schematic representation of spurious chromosome with 19 fragments corresponding to different wheat chromosomes and linkage of bin markers. (TIF)

S1 Table Bin marker segregation data and markers sequences of ‘Hewo’ x ‘Magnat’ genetic map. (XLSX)

S2 Table Bin markers order on ‘Hewo’ x ‘Magnat’ genetic map. (XLSX)

S3 Table Order of all markers mapped on ‘Hewo’ x ‘Magnat’ genetic map. (XLSX)
