# Supplementary figures and images for: Genetic Map of Triticale Integrating Microsatellite, DArT and SNP Markers
Source: PLoS One. 2015 Dec 30;10(12):e0145714. doi: 10.1371/journal.pone.0145714 (PMC4696847; doi:10.1371/journal.pone.0145714)

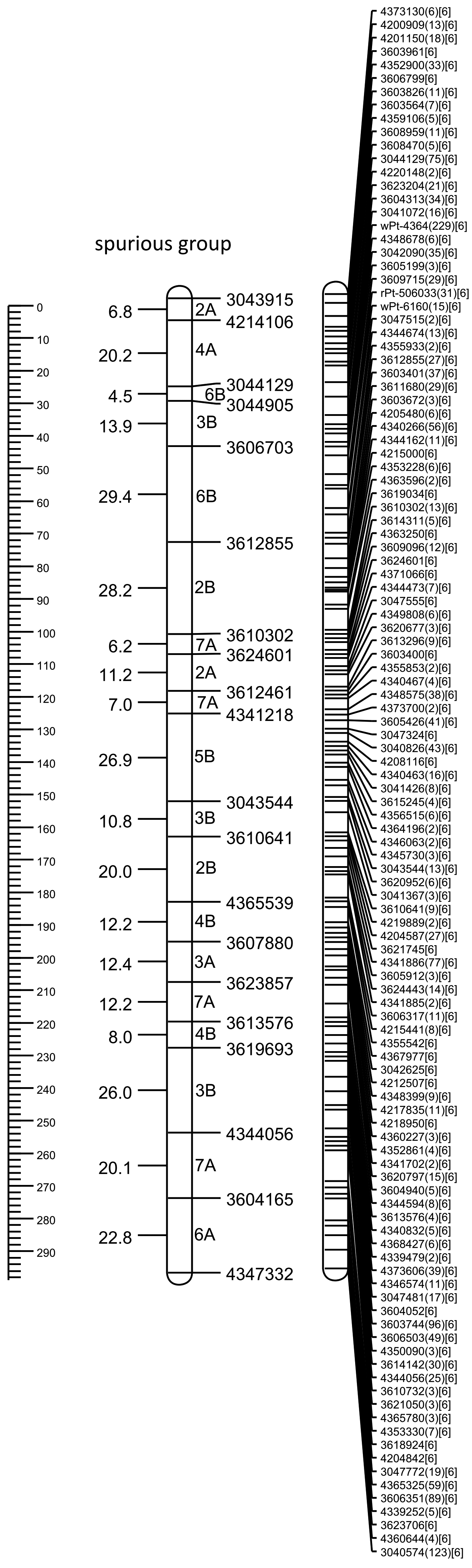

Supplement: S1 Fig — (TIF) [file pone.0145714.s001.tif]
